# Comparison of age‐dependent alterations in thioredoxin 2 and thioredoxin reductase 2 expressions in hippocampi between mice and rats

**DOI:** 10.1186/s42826-021-00088-y

**Published:** 2021-03-06

**Authors:** Yeon Ho Yoo, Dae Won Kim, Bai Hui Chen, Hyejin Sim, Bora Kim, Jae-Chul Lee, Ji Hyeon Ahn, Yoonsoo Park, Jun Hwi Cho, Il Jun Kang, Moo-Ho Won, Tae-Kyeong Lee

**Affiliations:** 1 Department of Emergency Medicine, Institute of Medical Sciences, School of Medicine, Kangwon National University Hospital, Kangwon National University, 24289 Chuncheon, Gangwon, Republic of Korea; 2 Department of Biochemistry and Molecular Biology, Research Institute of Oral Sciences, College of Dentistry, Gangnung-Wonju National University, 25457 Gangneung, Gangwon Republic of Korea; 3 Department of Histology and Embryology, Institute of Neuroscience, Wenzhou Medical University, 325035 Wenzhou, Zhejiang P.R. China; 4 Department of Neurobiology, School of Medicine, Kangwon National University, 24341 Chuncheon, Gangwon, Republic of Korea; 5 Department of Physical Therapy, College of Health Science, Youngsan University, 50510 Yangsan, Gyeongnam Republic of Korea; 6 Department of Food Science and Nutrition, Hallym University, 24252 Chuncheon, Gangwon, Republic of Korea; 7 Department of Biomedical Science and Research Institute for Bioscience and Biotechnology, Hallym University, 24252 Chuncheon, Gangwon, Republic of Korea

**Keywords:** Aging, Hippocampus, Rodent, Trx2, TrxR2

## Abstract

**Background:**

Aging is one of major causes triggering neurophysiological changes in many brain substructures, including the hippocampus, which has a major role in learning and memory. Thioredoxin (Trx) is a class of small redox proteins. Among the Trx family, Trx2 plays an important role in the regulation of mitochondrial membrane potential and is controlled by TrxR2. Hitherto, age-dependent alterations in Trx2 and TrxR2 in aged hippocampi have been poorly investigated. Therefore, the aim of this study was to examine changes in Trx2 and TrxR2 in mouse and rat hippocampi by age and to compare their differences between mice and rats.

**Results:**

Trx2 and TrxR2 levels using Western blots in mice were the highest at young age and gradually reduced with time, showing that no significant differences in the levels were found between the two subfields. In rats, however, their expression levels were the lowest at young age and gradually increased with time. Nevertheless, there were no differences in cellular distribution and morphology in their hippocampi when it was observed by cresyl violet staining. In addition, both Trx2 and TrxR2 immunoreactivities in the CA1-3 fields were mainly shown in pyramidal cells (principal cells), showing that their immunoreactivities were altered like changes in their protein levels.

**Conclusions:**

Our current findings suggest that Trx2 and TrxR2 expressions in the brain may be different according to brain regions, age and species. Therefore, further studies are needed to examine the reasons of the differences of Trx2 and TrxR2 expressions in the hippocampus between mice and rats.

## Background

The hippocampus in the brain is phylogenetically categorized as the archicortex and divided into three layers unlike the neocortex [1]. The hippocampus functionally belongs to the limbic system playing important roles in the consolidation of information from short term memory to long term memory, along with spatial memory and emotion [2–4]. It has been well acknowledged that neurophysiological changes due to pathological conditions, such as ischemic insults and neurodegenerative processes in the hippocampus, bring cognitive and memory dysfunctions [5–8].

Aging is an unavoidable process for almost living things and leads to detrimental changes in macromolecules and cells, which can elevate the risk of exposure to senile diseases [9]. Numerous studies have demonstrated that aging is one of major causes inducing neurochemical and neurophysiological alterations in various substructures of the brain, including the hippocampus [10–13]. Among the factors related with the aging process, reactive oxygen species (ROS) are regarded a key factor to engage brain aging [14, 15]. In particular, Petrosillo et al. (2008) have urged that taking into a consideration that the brain requires a high energy consumption, ROS-induced mitochondrial injuries can significantly advance aging process of the brain [15].

Thioredoxin, as a class of small redox proteins, is expressed in a large part of organisms and possesses dithiol-disulfide oxidoreductase activity [16]. Among the thioredoxin family, Trx2 plays a crucial role in the regulation of mitochondrial membrane potential and protection against ROS-induced apoptosis [17, 18]. Meanwhile, Trx2 activity is controlled by TrxR2 which is specifically presented in mitochondria and catalyzes oxidized Trx2 into reduced form [19, 20]. Therefore, along with Trx2, TrxR2 plays an pivotal role in maintaining the integrity of mitochondria by removing deleterious ROS [19].

Until now, studies regarding the roles of Trx2 and TrxR2 under pathophysiological conditions have been reported [21, 22]; however, age-dependent changes in Trx2 and TrxR2 in hippocampi of mice and rats have not been sufficiently examined and compared. Thus, the major objective of this study was to investigate changes of Trx2 and TrxR2 expressions in mouse and rat hippocampi and to compare them between mice and rats.

## Results

### Levels of Trx2 and TrxR2

**Fig. 1:**
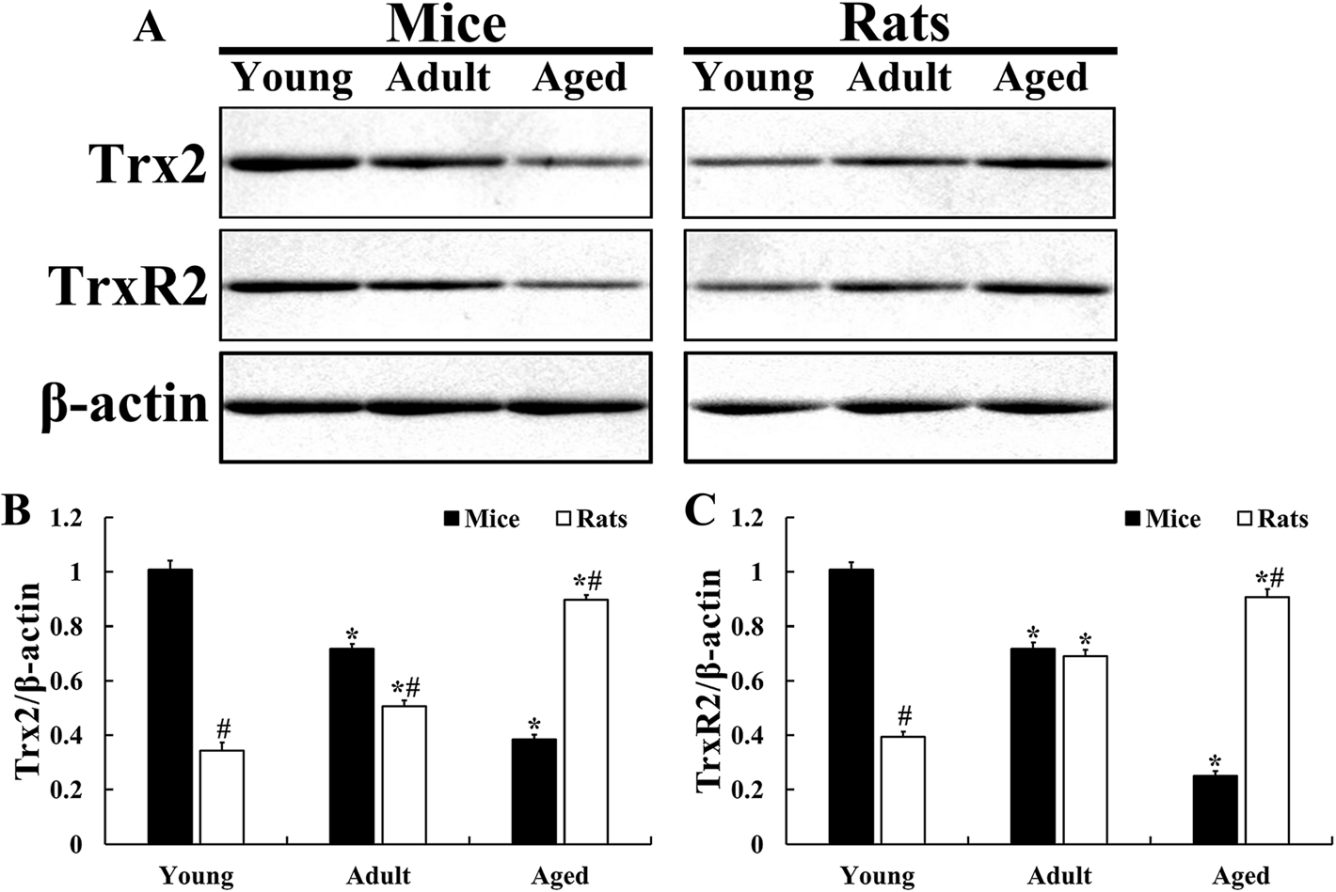
Representative immunoblots of Trx2 and TrxR2 (**a**) and normalized protein levels of Trx2 (**b**) and TrxR2 (**b**) versus each level of β-actin in the hippocampi of mice and rats. Expression levels of Trx2 and TrxR2 are decreased in the mice by age; contrastively, age-dependent increases of their expression levels are shown in the rats. The bars indicate the means ± SEM (*n* = 7/group; ^*^P < 0.05 versus mice at the same age or rats at early age; ^#^*P* < 0.05 versus mice at the same age)

Expression levels of Trx2 and TrxR2 in the mouse and rat hippocampus were examined by Western blot analysis. In the young mouse group, the protein levels of both Trx2 and TrxR2 were fundamentally detected in the hippocampus (Fig. 1a). The levels of Trx2 and TrxR2 in the adult mouse group were significantly decreased by 28.6 % and 28.7 % respectively and their levels were more significantly reduced in the aged mouse group by 61.9 % and 75.2 % respectively compared to those in the young mouse group (Fig. 1a , c).

In the young rat group, the levels of Trx2 and TrxR2 in the hippocampus were significantly lower (66.0 % and 61.0 % respectively) than those in the young mouse group (Fig. 1a, c). The protein levels of Trx2 and TrxR2 in the adult rat group were remarkably increased by 47.5 % and 75.1 % respectively, and, in the aged rat group, the protein levels were more significantly elevated by 161.4 % and 129.6 % respectively compared with those in the young rat group (Fig. 1a, c).

### Cresyl violet (CV)-stained cells

**Fig. 2:**
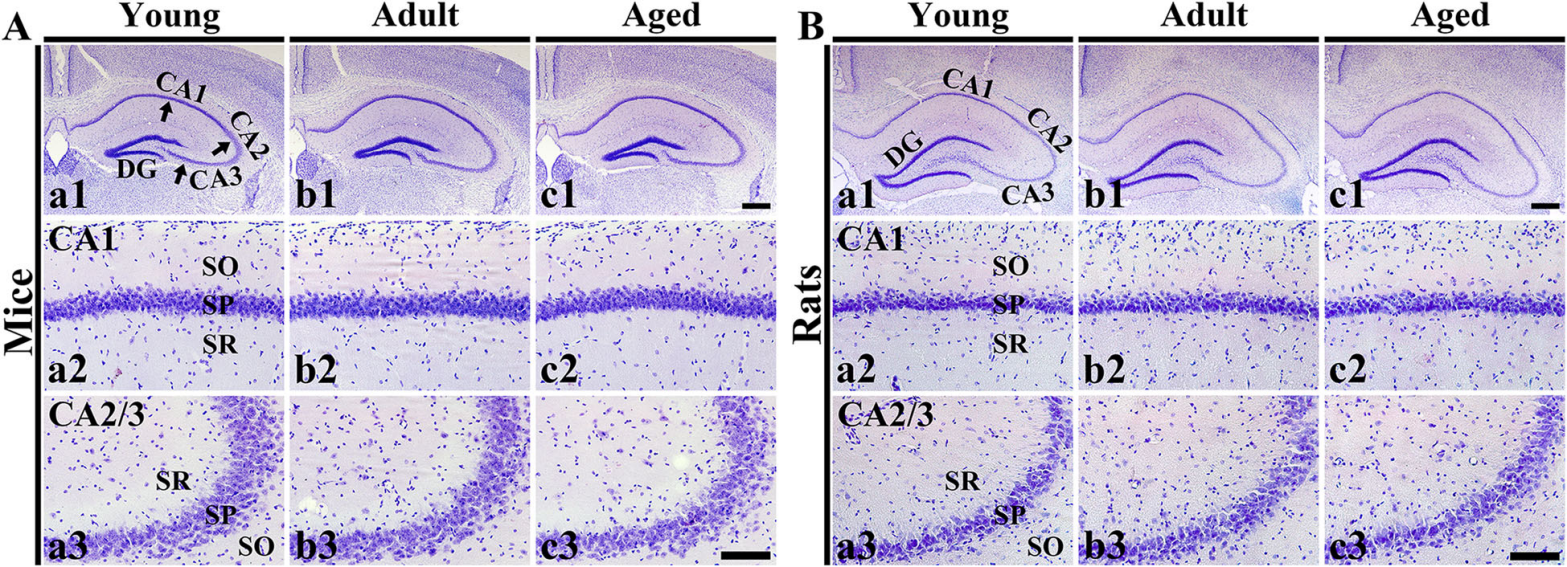
CV staining in mouse hippocampi (**a**; a1-c1) and rat hippocampi (**b**; a1-c1) containing the CA1 field (a2-c2) and CA2/3 field (a3-c3). Regardless of age and species, there are no significant differences in distribution pattern and morphology of CV-stained cells. Note that numerous CV-stained cells consist of stratum pyramidale (SP, arrows). DG, dentate gyrus; SO, stratum oriens; SR, stratum radiatum Scale bars = 400 µm (a1-c1) and 100 µm (a2-c2 and a3-c3) (*n* = 7/group)

The distribution pattern and cellular morphology in the mouse and rat hippocampus were observed by CV staining. In the young mice, cells in the CA1-3 fields were well stained by CV (Fig. 2 Aa1). In particular, numerous CV-stained cells formed stratum pyramidale (SP), in which cells are called pyramidal cells or neurons according to their morphology, in the CA1-3 fields (Fig. 2 Aa2 and Aa3). In the adult and aged mice, the distribution of CV-stained cells was not different from that of the young mice (Fig. 2 Ab1 and Ac1), showing that the morphology of the CV-stained cells was similar that in the young mice (Fig. 2 Ab2, Ac2, Ab3 and Ac3).

In the young rats, CV-stained cells in the CA1-3 fields were also easily observed like those in the young mice (Fig. 2 Ba1). In these, the morphology of the CV-stained pyramidal neurons was similar to that in the mice (Fig. 2 Ba2 and Ba3). In the adult and aged rats, the distributional pattern of CV-stained cells in the CA1-3 fields was also similar to that in the young rats (Fig. 2 Bb1 and BAc1), showing that their morphology was also similar to that in the young rats (Fig. 2 Bb2, Bc2, Bb3 and Bc3).

### Trx2 immunoreactivity

**Fig. 3:**
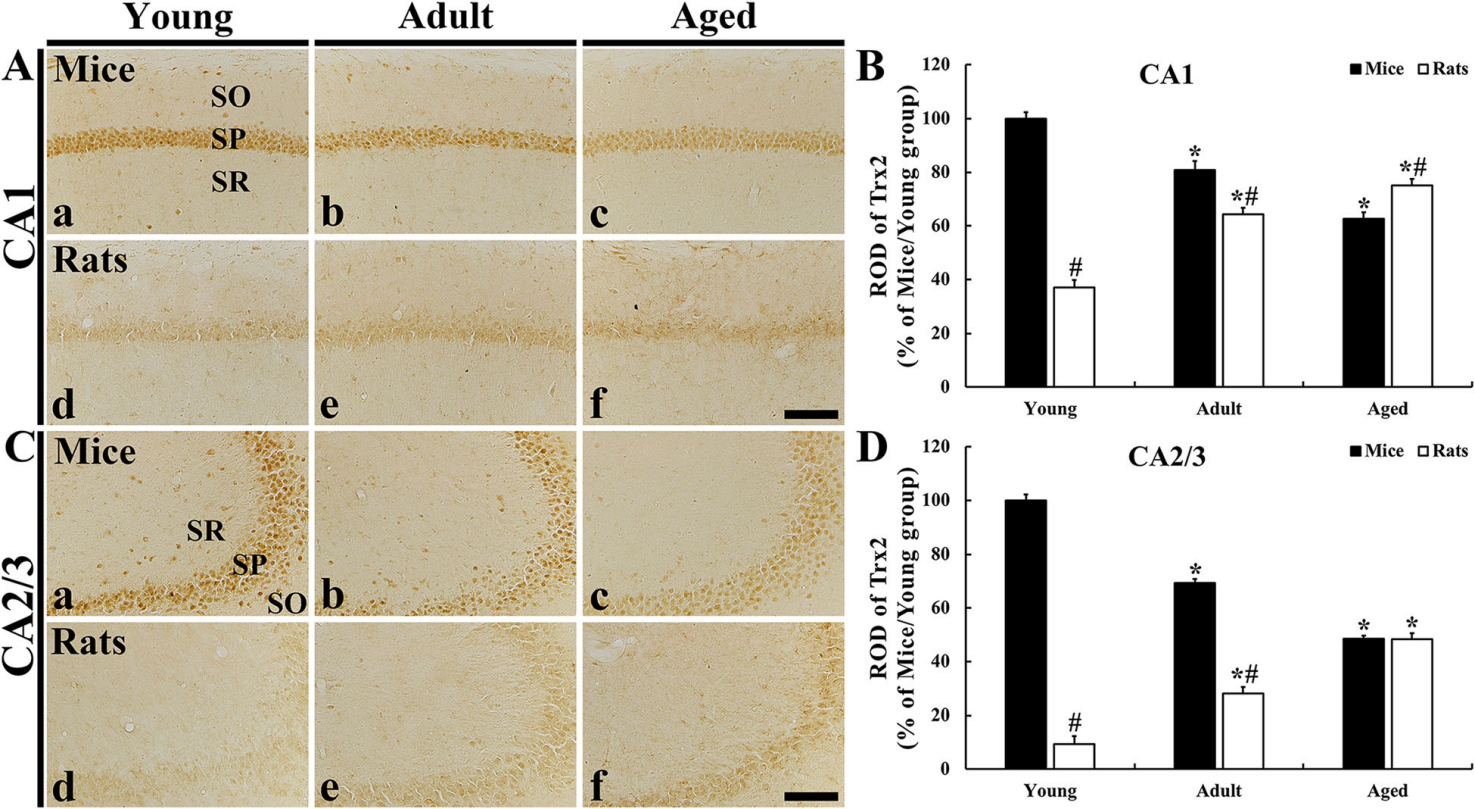
Immunohistochemistry for Trx2 in the CA1 (**a**) and CA2/3 (**c**) field of the mice (a-c) and rats (d-f). Trx2 immunoreactivity is principally shown in the SP of the CA1-3 fields. In the mice, Trx2 immunoreactivity is reduced with age, however, Trx2 immunoreactivity in the rats is increased age-dependently. **b** and **c**: ROD of Trx2 immunoreactivity in the CA1 (**b**) and CA2/3 (**c**) field. Scale bar = 100 µm. The bars indicate the means ± SEM (*n* = 7/group; ^*^P < 0.05 versus mice at the same age or rats at early age; ^#^*P* < 0.05 versus mice at the same age)

Immunohistochemistry for Trx2 was carried out in order to investigate age-dependent change in Trx2 immunoreactivity in the mouse and rat hippocampal subfields. In the young mouse group, Trx2 immunoreactivity were fundamentally found the SP in the CA1-3 fields, showing that significant difference in the distribution pattern of the Trx2 immunoreactivity was not found between the CA1 field and CA2/3 field (Fig. 3 Aa and Ca). In the adult mouse group, the ROD of Trx2 immunoreactivity in the CA1 and CA2/3 field was significantly decreased (80.9 % and 69.3 %, respectively, versus young mice) and more significantly reduced in the aged mice (CA1 and CA2/3, 62.6 % in CA1 and 48.5 % in CA2/2 versus young mice) compared with that in the young mouse group (Fig. 3 Ab, Ac, B, Cb, Cc and D).

In the young rat group, significantly low Trx2 immunoreactivity was detected (37.0 % in CA1 and 9.4 % in CA2/3 versus young mouse group) compared to that in the young mouse group (Fig. 3 Ad, B Cd and D). In the adult and aged rat group, Trx2 immunoreactivity in the CA1 and CA2/3 field was gradually increased with age, showing that ROD in the adult rat group was 79.5 % in the CA1 field and 40.5 % in the CA2/3 field compared with that in the adult mouse group, and ROD in the aged rat group was 120.0 % in the CA1 field and 99.9 % in the CA2/3 filed compared with that in the aged mouse group (Fig. 3 Ae, Af, B, Ce and Cf and D).

### TrxR2 immunoreactivity

**Fig. 4:**
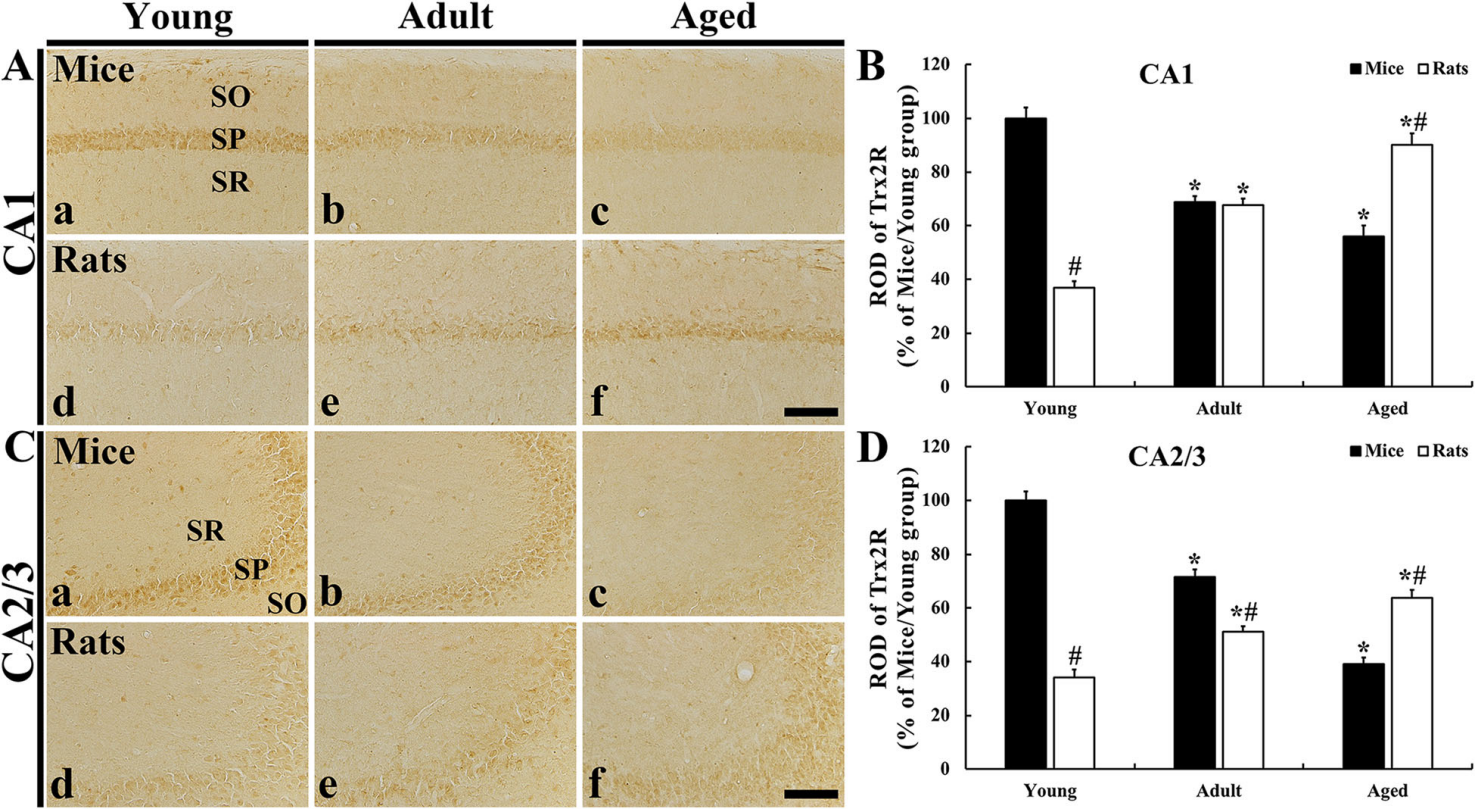
Immunohistochemistry for TrxR2 in the CA1 (**a**) and CA2/3 (**c**) field of the mice (a-c) and rats (d-f). TrxR2 immunoreactivity is typically shown in the SP. In the mice, age-dependent decrease of TrxR2 immunoreactivity is shown in the SP. Contrastively, TrxR2 immunoreactivity in the rats is gradually increased with age. **b** and **c**: ROD of TrxR2 immunoreactivity in the CA1(**b**) and CA2/3 (**c**) field. Scale bar = 100 µm. The bars indicate the means ± SEM (*n* = 7/group; ^*^P < 0.05 versus mice at the same age or rats at early age; ^#^*P* < 0.05 versus mice at the same age)

To examine age-dependent alteration in TrxR2 immunoreactivity in the mouse and rat hippocampal subfields, immunohistochemistry for TrxR2 was performed. In the young mice, TrxR2 immunoreactivity was also mainly in the SP of the CA1 and CA2/3 field like Trx2 immunoreactivity in the young mice (Fig. 4 Aa and Ca). In the adult mice, TrxR2 immunoreactivity in the CA1 and CA2/3 field was significantly reduced (68.8 % in CA1 and 71.6 % in CA2/3 versus the young mice), and, in the aged mice, TrxR2 immunoreactivity was more significantly decreased (56.0 % in CA1 and 39.1 % in CA2/3 versus the young mice) compared to that in the young mice (Fig. 4 Ab, Ac, B, Cb, Cc and D).

In the young rats, significantly low TrxR2 immunoreactivity was found (36.9 % in CA1 and 34.0 % in CA2/3 versus young mice) compared to that in the young mice (Fig. 4 Ad, B Cd and D). In the adult and aged rats, TrxR2 immunoreactivity in the CA1 and CA2/3 field was gradually increased with age, showing that ROD in the adult rats was 98.4 % in the CA1 field and 71.4 % in the CA2/3 field compared with that in the adult mice, and, in the aged rats, ROD was 161.0 % in the CA1 field and 163.1 % in the CA2/3 field compared with that in the aged mice (Fig. 4 Ae, Af, B, Ce and Cf and D).

## Discussion

Many studies have reported age-dependent alterations of various factors in rodent brains. For instance, it was reported that p53 and p63 are reduced age-dependently in mouse hippocampus, which might be closely involved in cellular senescence [12]. Ahn et al. (2019) demonstrated that, in gerbil hippocampus, expression of nuclear receptor related-1 protein, which is considered as deeply connected with cognitive function, was reduced with the advance of age [13]. In addition, Lee et al. (2018) showed age-dependent decrease in the expressions of insulin-like growth factor (IGF) I and its receptor in gerbil olfactory bulb [11]. Moreover, there is a comparative study showing that various changes of calcium-binding proteins including calbindin-D28k, calretinin and parvalbumin in the somatosensory cortex between mice, rats and gerbils [10]. The above precedent studies show that those factors in the rodent brains are altered in a regular pattern with age.

Our current examination of CV staining data revealed that, regardless of age or rodent species, there was no significant difference in cellular distributions and morphology in the CA1-3 fields between mice and rats. Interestingly, however, our current immunoblot results showed an opposite pattern in age-dependent changes of Trx2 and TrxR2 protein levels between the mouse and rat hippocampus. Namely, the levels of Trx2 and TrxR2 proteins were age-dependently decreased in the mouse hippocampus, whereas they were age-dependently increased in the rat hippocampus. Based on the immunoblot analysis result, immunohistochemical stainings for Trx2 and TrxR2 in the CA1-3 fields were carried out to examine each immunoreactive intensity in hippocampal subfields (CA1-3 fields) and found that Trx2 and TrxR2 immunoreactivity was essentially detected in pyramidal cells, which are principal neurons in the CA1-3 fields, and the change pattern of each immunoreactivity was identical to the change pattern seen in the Western blots. Unfortunately, to the best of our knowledge, we cannot explain the reason of age-dependent differences in Trx2 and TrxR2 expressions in the hippocampus between mice and rats. Therefore, further studies regarding to the different age-dependent changes in Trx2 and TrxR2 in the mouse and rat hippocampus are needed.

A number of studies have proven the roles of Trx2 and TrxR2 in cell survival under some insults. For example, Huang et al. (2015) have reported that Trx2 inhibits mitochondrial ROS generation and apoptosis stress kinase-1 activity to maintain cardiac function in cardiac-specific Trx2 knockout mice [23]. In particular, Li et al. (2017) have demonstrated that Trx2 offers protection against mitochondrial oxidative stress in high glucose-treated H9c2 cardiac cells and against myocardial hypertrophy induced by hyperglycemia in streptozotocin (STZ)-induced diabetic rats [24].

In brains, it has been addressed that Trx2 and TrxR2 exert crucial effects on neuronal survival under brain insults. Lee et al. (2017) have demonstrated that ischemic preconditioning offers neuroprotective effect in the hippocampus by maintaining Trx2 and TrxR2 expressions in a gerbil model of transient forebrain ischemia [22]. In that study, the neuroprotective effect mediated by ischemic preconditioning was abolished when the animals were administered auranofin (an inhibitor of TrxR2) [22]. In addition, it has been reported that aged gerbils display much delayed neuronal death in pyramidal neurons located in the CA1 field following 5-min transient forebrain ischemia compared to that in adult gerbils [25]. In the study, the expression level of Trx2 in the CA1 field of aged gerbils is significantly higher than that in adult ones after the ischemic insult [26].

## Conclusions

In summary, there was no difference in cellular distribution and morphology between the mouse and rat hippocampus. However, Trx2 and TrxR2 expressions were reduced in mouse hippocampus with age; whereas, in rat hippocampus, their expressions were age-dependently increased, although Trx2 and TrxR2 immunoreactivity in both hippocampi was shown in pyramidal cells (principal neurons). With respect to our current findings, further studies are needed to explain the reason of age-dependent difference in Trx2 and TrxR2 expression in the hippocampus between mice and rats.

## Methods

### Experimental animals and groups

Male ICR mice (*n* = 42) and male Sprague-Dawley rats (*n* = 42) were used. They were obtained from the Experimental Animal Center installed in Kangwon National University (Chuncheon, Kangwon, Korea). The animals were housed under conventional conditions which were established with suitable room temperature (23 ± 1℃) and relative humidity (55 ± 5 %). We provided constant 12-h period of dark-light cycle and liberally accessible feeds and water to the animals. The protocol of this research was authorized (approval No., KW-180124-2; approval date, May 22, 2018) by Institutional Animal Care and Use Committee under the President of Kangwon National University. The protocol devoted the guidelines of the “Current International Laws and Policies”, a part of the “Guide for the Care and Use of Laboratory Animals” [27].

The animals were grouped into six groups (*n* = 14/group) according to their age: (1) young mice at 1 month, (2) adult mice at 6 months, (3) aged mice at 24 months, (4) young rats at 1 month, (5) adult rats at 6 months, and (6) aged rats at 24 months.

### Western blot

With reference to some previous studies [28, 29], Western blot analyses for Trx2 and TrxR2 were carried out. In brief, seven animals per group were deeply anesthetized according to a methodology of anesthesia [30]. In detail, 60 mg/kg pentobarbital sodium (JW pharm. Co., Ltd., Seoul, Republic of Korea) was intraperitoneally injected for the mice, and intraperitoneal administration with a mixture of 100 mg/kg ketamine hydrochloride (Virbac Korea Co., Ltd., Seoul, Korea) and 10 mg/kg Rompun® (xylazine) (Bayer Korea Ltd., Seoul, Korea) was done for the rats. Their brains were removed and homogenize with 50 mM phosphate-buffered saline (pH 7.4) containing 0.1 mM ethylene glycol-bis (β-aminoethyl ether)-N,N,N′,N′-tetraacetic acid (EGTA) (pH 8.0), 10 mM ethylenediaminetetraacetic acid (EDTA) (pH 8.0), 0.2 % Nonidet P-40, 15 mM sodium pyrophosphate, 100 mM β-glycerophosphate, 2 mM sodium orthovanadate, 50 mM NaF, 150 mM NaCl, 1 mM phenylmethylsulfonyl fluoride (PMSF), and 1 mM dithiothreitol (DTT). The homogenized brain tissues were centrifuged, and the supernatants were taken to evaluate protein levels using a Micro BCA assay kit of Thermo Fisher Scientific Inc (MA, USA) with bovine serum albumin (Pierce Chemical, Rockford, IL, USA) as a standard. Aliquots including 20 µg of total protein were boiled in a loading buffer containing 150 mM Tris (pH 6.8), 6 % SDS, 3 mM DTT, 0.3 % bromophenol blue, and 30 % glycerol. The samples were separated by 10 % SDS-PAGE. Subsequently, the gels were transferred to nitrocellulose membranes of Pall Co (East Hills, NY, USA) at 350 mA and 4℃ for 100 min. The membranes were incubated with a 5 % skimmed milk to block non-specific staining at room temperature for one hour. Thereafter, they were immunoreacted with each primary antibody for 1 day at 4℃. The primary antisera were rabbit anti-Trx2 (diluted 1:5,000) (A-Frontier Co., Ltd., Seoul, Korea) and rabbit anti-TrxR2 (diluted 1:2,000) (A-Frontier Co., Ltd., Seoul, Korea) and rabbit anti-β-actin (diluted 1:2,000) (Sigma-Aldrich, St. Louis, MO, USA). Thereafter, each membrane was incubated with HRP-conjugated donkey anti-rabbit IgG (diluted 1:5,000) (Santa Cruz Biotechnology, Santa Cruz, CA, USA) for 1 h at room temperature. Finally, luminol-based chemiluminescence kit from Pierce (Thermo Fisher Scientific Inc., MA, USA) was used to enhance visualization.

Immunoblots of Trx2 and TrxR2 were analyzed according to previously described method [31] with minor modification. In short, using Scion Image software from Scion Crop (Frederick, MD, USA), the bands were respectively scanned, and densitometric analysis was performed. The protein levels were respectively normalized versus the corresponding level of β-actin.

### Preparation of histological sections

For histological investigation, the sections of the brains containing the hippocampi were prepared according to previously published methods [32, 33]. Seven animals per group were deeply anesthetized according to an established anesthetic methodology [30]. Namely, 60 mg/kg pentobarbital sodium (JW pharm. Co., Ltd., Seoul, Korea) for the mice, and a mixture of 100 mg/kg ketamine hydrochloride (Virbac Korea Co., Ltd., Seoul, Korea) and 10 mg/kg Rompun® (xylazine) (Bayer Korea Ltd., Seoul, Korea) was intraperitoneally injected. Under anesthetic condition, their brains were rinsed by transcardial perfusion with saline and fixed with 4 % paraformaldehyde solution (in 100 mM PB, pH 7.4). The fixed brains were harvested and stored in the same fixative for 5 h at room temperature. To make the brain sections, the fixed brains were put in 30 % sucrose solution (in 100 mM PB, pH 7.4) for 1 day at room temperature to avoid the brains from freezing damage. Thereafter, the brains were coronally cut on sliding microtome of SM2010 R (Leica Nussloch, Germany) equipped with BFS-40MP freezing stage (Physitemp Instruments, Inc., NJ, USA) at 30-µm thickness. These sections were stored in six wells plates containing 100 mM PBS (pH 7.4) in the dark at 4ºC.

### CV staining

CV staining was carried out with reference to a method by Zhu et al. (2015) [34]. In short, the sections were stained with solution of 0.1 % (*w/v*) CV acetate (Sigma-Aldrich Co., St. Louis, MO, USA) for 25 min at room temperature. Thereafter, the CV-stained sections were rinsed in distilled water and discolored in 50 % ethyl alcohol for a few seconds, and these sections then were dehydrated by immersing into serial ethyl alcohol baths (70 %, 80 %, 90 %, 95 % and 100 %) for 5 min, respectively. Finally, these sections were cleared in xylene for 3 min and mounted with Canada balsam from Kanto Chemical Co., Inc. (Tokyo, Japan).

The images of CV-stained cells in the hippocampi were captured at magnifications of 4X (for lower magnification) and 20X (for higher magnification) using light microscope (BX53) from Olympus (Tokyo, Japan) equipped with digital camera (DP72) (Olympus, Tokyo, Japan) and analyzed the captured images using microscopy imaging software of cellSens Standard (Olympus, Tokyo, Japan).

### Immunohistochemistry

To examine changes in Trx2 and TrxR2 immunoreactivity in the hippocampi, avidin-biotin complex (ABC) method was used with reference to previously described methods [34, 35] with minor modification. The sections were rinsed with 100 mM PBS (pH 7.4) three times, and an activity of endogenous peroxidase in the tissues was blocked with 0.3 % hydrogen peroxidase (H_2_O_2_) solution for 20 min at room temperature, in succession, non-specific proteins were blocked by immersing them into 5 % normal goat serum (in saline) for 30 min at room temperature. Next, the sections were immunoreacted with primary antibodies: rabbit anti-Trx2 (1:500 in 100 mM PBS; A-Frontier Co., Ltd., Seoul, Korea) and rabbit anti-TrxR2 (same as Trx2) for 8 h at 4℃. Thereafter, the sections were rinsed with 100 mM PBS and reacted in biotinylated goat anti-rabbit IgG (1:250 in 100 mM PBS; Vector Laboratories, Burlingame, CA, USA) followed by ABC (1:300 in 100 mM PBS; same as IgG) for 120 min and 60 min, respectively, at room temperature. Finally, the sections were visualized by putting in solution of 0.06 % 3, 3’-diaminobenzidine tetrahydrochloride (DAB) from Sigma-Aldrich Co (St. Louis, MO, USA) in 100 mM PBS containing 0.1 % H_2_O_2_. Finally, the sections were rinsed in PBS, dehydrated in serial ethyl alcohol (70 %, 80 %, 90 %, 95 % and 100 %), cleared in xylene and coverslipped with Canada balsam. In addition, for negative control test, the same brain sections were reacted with the pre-immune serum except for the primary antibodies, and no immunoreactive structures were detected in the tested sections (data not shown).

To quantify Trx2 and TrxR2 immunoreactivity in the CA1 and CA2/3 field, the optical density of each immunoreactivity was presented. Five brain sections per animal were selected, and each immunoreactivity was captured using light microscope (BX53) from Olympus (Tokyo, Japan) with digital camera (DP72) of Olympus (Tokyo, Japan) and analyzed with image capture software (cellSens Standard) from Olympus (Tokyo, Japan). With reference to a method (Paizs et al., 2009), colored images were captured and converted into 8-bit grey scale images with a range of 0-255 (from black to white) [36]. The images were evaluated for grey scale intensity and the immunoreactive intensities of immunoreaction within the area of interest were computed using Image J software (version 1.46) from National Institutes of Health (Bethesda, MD, USA). The immunoreactive intensities were presented as relative optical density (ROD). The ROD of each young group was considered as 100 %.

### Statistical analysis

Using GraphPad Prism software (version 5.0; GraphPad Software, La Jolla, CA, USA), all data were statistically analyzed. Data obtained from the current experiments were presented as the mean ± standard error of mean (SEM). The statistical significances of the mean among the experimental groups were determined by two-way analysis of variance (ANOVA) with a *post hoc* Bonferroni’s test for all pairwise multiple comparisons. By lower than 0.05 of P value, the significant differences were designated.

## Data Availability

All data produced and analyzed in the current study are included in this paper.

## Abbreviations

CA: Cornu ammonis
CV: Cresyl violet
Trx2: Thioredoxin 2
TrxR2: Thioredoxin reductase 2
